# Cats and dogs: A mesopredator navigating risk and reward provisioned by an apex predator

**DOI:** 10.1002/ece3.8641

**Published:** 2022-02-22

**Authors:** Mitchell J. Brunet, Kevin L. Monteith, Katey S. Huggler, Justin G. Clapp, Daniel J. Thompson, Patrick W. Burke, Mark Zornes, Patrick Lionberger, Miguel Valdez, Joseph D. Holbrook

**Affiliations:** ^1^ Haub School of Environment and Natural Resources University of Wyoming Laramie Wyoming USA; ^2^ Wyoming Cooperative Fish and Wildlife Research Unit University of Wyoming Laramie Wyoming USA; ^3^ Department of Zoology and Physiology University of Wyoming Laramie Wyoming USA; ^4^ Wyoming Game and Fish Department Lander Wyoming USA; ^5^ Wyoming Game and Fish Department Green River Wyoming USA; ^6^ Bureau of Land Management Rock Springs Field Office Rock Springs Wyoming USA

**Keywords:** coyote, encounter rate, intraguild predation, mountain lion, predation risk, scavenge

## Abstract

Successfully perceiving risk and reward is fundamental to the fitness of an animal, and can be achieved through a variety of perception tactics. For example, mesopredators may “directly” perceive risk by visually observing apex predators, or may “indirectly” perceive risk by observing habitats used by predators. Direct assessments should more accurately characterize the arrangement of risk and reward; however, indirect assessments are used more frequently in studies concerning the response of GPS‐marked animals to spatiotemporally variable sources of risk and reward. We investigated the response of a mesopredator to the presence of risk and reward created by an apex predator, where risk and reward likely vary in relative perceptibility (i.e., degree of being perceptible). First, we tested whether coyotes (*Canis latrans*) use direct or indirect assessments to navigate the presence of mountain lions (*Puma concolor*; risk) and kills made by mountain lions (reward) in an area where coyotes were a common prey item for mountain lions. Second, we assessed the behavioral response of coyotes to direct encounters with mountain lions. Third, we evaluated spatiotemporal use of carrion by coyotes at kills made by mountain lions. Indirect assessments generally outperformed direct assessments when integrating analyses into a unified framework; nevertheless, our ability to detect direct perception in navigating to mountain lion kills was likely restricted by scale and sampling limitations (e.g., collar fix rates, unsampled kill sites). Rather than responding to the risk of direct encounters with mountain lions, coyotes facilitated encounters by increasing their movement rate, and engaged in risky behavior by scavenging at mountain lion kills. Coyotes appear to mitigate risk by using indirect perception to avoid mountain lions. Our predator–predator interactions and insights are nuanced and counter to the conventional predator–prey systems that have generated much of the predation risk literature.

## Graphical abstract

Successfully perceiving risk and reward can be achieved through direct or indirect cues. First, we tested whether coyotes (Canis latrans) use direct or indirect assessments to navigate the presence of mountain lions (Puma concolor; risk) and kills made by mountain lions (reward) in an area where coyotes were a common prey item for mountain lions. Second, we assessed the behavioral response of coyotes to direct encounters with mountain lions. Third, we evaluated spatiotemporal use of carrion by coyotes at kills made by mountain lions.

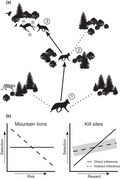

## 1 INTRODUCTION

An animal survives through its ability to balance risk and reward. Risk is an expression of the fitness consequences that an animal faces, whereas reward represents resources that an animal may use to increase its fitness (Steinhoff et al., 2020). The ability to shift behavior is an essential component in balancing risk and reward; however, a behavioral response is conditioned on what an animal is able to perceive (Gaynor et al., 2019; Prugh et al., 2019). Perception occurs through sensory mechanisms, whereby an animal is able to detect a cue from its environment, and ultimately, the perception of that cue can trigger a behavioral response (Makin et al., 2019; Parsons et al., 2018). The perceptibility (i.e., degree of being perceptible) of cues varies not only because of their source but also because of variation in the sensory capabilities of the animal that detects and responds to them (Jordan & Ryan, 2015).

Responses to risk and reward are well studied across taxa (Jurcak & Moore, 2018; van der Meer et al., 2012; Suraci et al., 2016; Wilmers et al., 2003; Zanette et al., 2011); however, these responses are contingent upon perception of associated cues, which is less understood (Jordan & Ryan, 2015; Prugh et al., 2019). If an animal perceives and responds to a cue coming from, or directly associated with a source of risk or reward, then we can consider the response to be the result of direct perception. For instance, turkey vultures (*Cathartes aura*) almost exclusively locate carrion by keying into olfactory cues in the air (Grigg et al., 2017; Houston, 1986), and other scavengers likely associate the visual cues of feeding vultures with the presence of food resources (Buckley, 1996; Pöysä, 1992). Conversely, if an animal is unable to perceive a cue directly, but responds to another cue indirectly related to the source (i.e., habitat features), then the response is the result of indirect perception. Indirect perception is likely conditioned on prior experiences that involved direct cues. Through memory or learning, an animal may begin to associate habitat features or particular areas with that used by a predator (Creel, 2018).

Risk and reward are represented indirectly in many studies involving GPS‐marked animals by developing a model that links habitat features to risk and reward, which is then applied across the landscape to generate spatial predictions. For example, the risk of encountering predators within predator–prey systems often is predicted based on habitat features used by predators (Atwood et al., 2009; Ditmer et al., 2018; Hebblewhite et al., 2005; Mumma et al., 2017), rather than by the actual location of predators at a given point in time. Researchers estimate habitat selection for a predator, and then generate a map of relative risk based on the habitat features occurring on the landscape. Indirectly indexing the location of risk and reward, however, has potentially led to weakened inference relative to more direct assessments (Prugh et al., 2019). A direct assessment may be favored when an animal is capable of perceiving the location of risk or reward on the landscape, especially when the position of risk or reward is spatiotemporally variable, because a direct assessment clearly conveys the presence of risk and reward itself, and more accurately characterizes their true arrangement. Alternatively, indirect assessments may be favored, especially when avoiding the risk of predation, if they improve an animal's ability to avoid detection by a predator (Creel, 2018). In these cases, even if an animal does not know the true location of a predator, the outcome is likely more favorable if an encounter is avoided altogether. In addition, if an animal is unable to perceive risk or reward directly, then its true arrangement at a given point in time is unlikely to influence how an animal navigates the landscape, and indirect assessments may outperform direct assessments in characterizing movement behaviors.

A mesopredator navigating space shared by an apex predator should attempt to identify the presence of risk imposed by the apex predator. Apex predators can suppress mesopredator populations through competition (Berger & Gese, 2007; Petroelje et al., 2021) and direct killing (Palomares & Caro, 1999), and also can influence behavior through nonconsumptive effects (Ritchie & Johnson, 2009). Intraguild predation occurs when one predator species kills, and at least partially consumes another, with whom it may compete for shared resources (Holt & Polis, 1997; Lourenco et al., 2014; Polis et al., 1989). If predation risk poses a considerable threat to a mesopredator, then the mesopredator should implement avoidance strategies to mitigate risk. In contrast, mesopredators frequently scavenge carrion left at kill sites made by apex predators (Moleón et al., 2014; Sivy et al., 2018; Wilson & Wolkovich, 2011), despite the potential risk associated with navigating those areas (Prugh & Sivy, 2020). Scavenging is increasingly recognized as a successful foraging strategy for mesopredators (Prugh et al., 2019; Ruprecht et al., 2021; Sivy et al., 2017, 2018), suggesting there may be opposing attraction and avoidance based on the risk and reward created by apex predators. As such, interactions within the predator guild may be unique with respect to risk, compared with more traditional predator–prey studies (e.g., Ditmer et al., 2018; Hebblewhite et al., 2005; Kohl et al., 2018; Périquet et al., 2012). Variation in the strength of sensory cues associated with the risk and reward created by apex predators makes interactions within the predator guild a compelling system to test direct and indirect assessments. Moreover, how a mesopredator perceives and responds to risk and reward has important carryover effects on its use of the landscape, and accordingly, the spatiotemporal arrangement and magnitude of predation risk it imposes on its own subordinates or prey (Gordon et al., 2015).

We investigated how a mesopredator navigates the risk–reward landscape created by an apex predator, through the lens of coyotes (*Canis latrans*) with respect to mountain lions (*Puma concolor*). Coyotes and mountain lions use overlapping home ranges, similar habitat and prey (Hurley et al., 2011; Koehler & Hornocker, 1991; Pierce et al., 2005), and intraguild killing of coyotes by mountain lions can be common (Boyd & O’Gara, 1985; Elbroch & Kusler, 2018; Knopff et al., 2010; Lowrey et al., 2016; Ruprecht et al., 2021). Coyotes are frequent scavengers and target carrion left by other predators (Atwood & Gese, 2010; Atwood et al., 2009; Koehler & Hornocker, 1991; Mahoney, 2017; Ruprecht et al., 2021), in some instances driving more dominant predators off of kill sites (Atwood & Gese, 2008). Mountain lions, therefore, provide not only predation risk (Koehler & Hornocker, 1991) but also reward in the form of carrion (Allen et al., 2014; Elbroch & Wittmer, 2012; Ruprecht et al., 2021) to coyotes that successfully navigate the risk–reward landscape.

We evaluated three questions concerning how coyotes respond to direct and indirect features associated with mountain lions and carrion found at kills made by mountain lions. First, we tested whether coyotes selected or avoided mountain lions and kills made by mountain lions based on direct or indirect assessments of their presence on the landscape. Being Canids, coyotes should be well equipped to locate carrion directly based on the presence of strong olfactory and visual cues (Danner & Smith, 1980; Kamler et al., 2004; Natusch et al., 2017), but coyotes may be limited in their ability to detect mountain lions. Mountain lions are stalk‐and‐ambush predators (Beier et al., 1995), and should be difficult to perceive directly, but should occupy somewhat predictable habitats (Makin et al., 2017; Preisser et al., 2007) and thus be more perceptible via indirect means. Coyotes navigating risky areas may be better equipped to identify habitats (i.e., indirect assessment) frequented by mountain lions, than the exact location of a mountain lion itself (i.e., direct assessment). Based on relative perceptibility, we hypothesized coyotes would indirectly avoid habitats used most by mountain lions, while directly accessing the reward of carrion through direct cues. In our second question, we evaluated the response of coyotes to direct encounters with mountain lions. We tested movement behavior and habitat selection by coyotes, hypothesizing that because of the low perceptibility of mountain lions, coyotes would not respond to their presence during encounters. We predicted coyote movement and habitat selection would remain unaltered pre‐ and post‐encounter with mountain lions. Finally, we assessed coyote use of kills made by mountain lions, hypothesizing that coyotes would identify and scavenge carrion left by mountain lions. We predicted coyote use at kill sites would increase with time, and then decline, consistent with a discovery phase, followed by scavenging and then vacancy.

## 2 MATERIALS AND METHODS

### 2.1 Study site

We conducted our research in the Greater Little Mountain Area (6400 km^2^; 41°05'49.9"N 109°17'18.1"W) located in southwestern Wyoming, USA. Low elevation (~1800 m) valleys in the study area were mixed sage‐grassland (*Artemesia* spp., blue‐bunch wheatgrass [*Pseudoroegneria spicata*] and cheatgrass [*Bromus tectorum*]), transitioning into sage and pinyon–juniper (*Juniperus* spp.) at intermediate elevations (~2400 m), and to occasional stands of quaking aspen (*Populus tremuloides*) and subalpine fir (*Abies lasiocarpa*) with mixed‐shrub understory at high elevations (~2700 m). Dominant shrub species throughout the study area included antelope bitterbrush (*Purshia tridentata*), big sagebrush (*A*. *tridentata*), mountain mahogany (*Cercocarpus montanus*), and wax currant (*Ribes cereum*).

Coyotes and mountain lions were the two largest predators on the landscape, excluding black bears (*Ursus americanus*), which were low in abundance. Mule deer (*Odocoileus hemionus*), elk (*Cervus canadensis*), and pronghorn (*Antilocapra americana*) were the most abundant ungulates, followed by moose (*Alces alces*) and a small population of bighorn sheep (*Ovis canadensis*) on the southern edge of the study area. Mountain lion diets consisted primarily of mule deer (63.4%), elk (12.2%), and pronghorn (11.5%; Clapp et al., in press). Coyotes accounted for the next largest percentage of the total 262 mountain lion kills (8.4%), indicating the risk of mortality to coyotes from mountain lions was higher compared with other systems where coyotes were absent or present at lower levels in mountain lion diets (Anderson & Lindzey, 2003; Elbroch et al., 2013; Knopff et al., 2010; Wilckens et al., 2016).

### 2.2 Animal captures and monitoring

We captured coyotes using foothold traps (*n* = 3) and helicopter netgunning (*n* = 36) during April 2017–April 2019. We fitted coyotes with GPS collars (Advanced Telemetry Systems Inc., Isanti, Minnesota, USA) programmed to acquire one location every hour during 15 May–30 September and one location every eight hours during 1 October–14 May. We captured mountain lions using hounds, baited cage traps, and foothold traps (*n* = 6) during May 2016–April 2018. We immobilized mountain lions using a combination of tiletamine and zolazepam (Telazol, Zoetis Inc., Kalamazoo, Michigan; 4.84 mg/kg) and xylazine (Anased, LLOYD Inc., Shenandoah, Iowa; 0.99 mg/kg) and administered 2.0 mg/kg tolazoline hydrochloride as a reversal agent after processing each animal. We fitted mountain lions with GPS collars (Telonics Inc., Mesa, Arizona, USA) programmed to acquire one location every hour during 15 June–15 July and one location every three hours during 16 July–14 June.

We conducted animal captures in accordance with guidelines from the American Society of Mammalogists (Sikes, 2016), and in compliance with University of Wyoming Animal Care and Use Committee (protocol number 20170404KM00270), and the Wyoming Game and Fish Department.

### 2.3 Analytical overview

**FIGURE 1:**
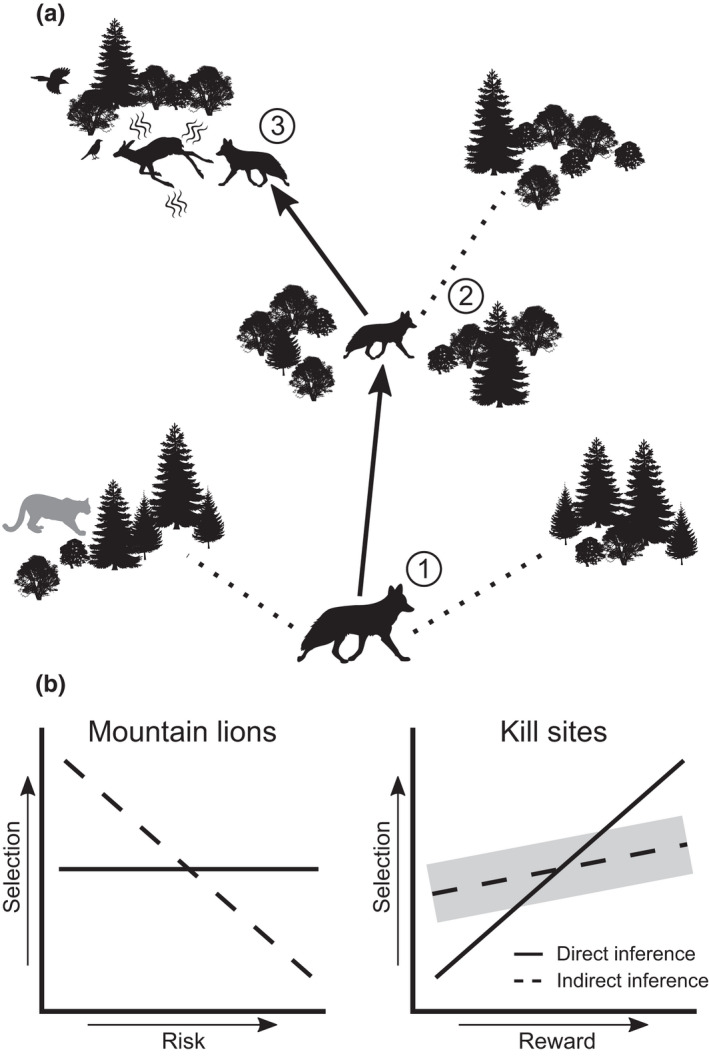
Conceptual figure of the predictions associated with navigating sources of risk and reward that vary in perceptibility. (a) (1) A coyote (*Canis latrans*) navigating the risk associated with mountain lions (*Puma concolor*) should use indirect cues based on the habitat features associated with mountain lions to mitigate the risk of predation. (2) A coyote navigating the reward provided by carrion at kill sites may initially use indirect cues to search areas associated with carrion left by mountain lions. (3) A coyote that reaches the perceptive range of a kill made by a mountain lion should navigate toward it based on direct cues coming from the kill. (b) When we assess selection by coyotes for areas or habitats used by mountain lions, we should observe strong avoidance associated with increasing risk. When assessed directly, we predict that selection by coyotes will not be associated with the presence of mountain lions, because perception should limit the ability for a coyote to respond to the actual location of a mountain lion. When reward from kills made by mountain lions is assessed indirectly, coyotes should show some, albeit variable (gray ribbon), selection for kill sites if they occur in predictable areas. However, coyotes should show stronger selection when reward is assessed directly, because kill sites should be easily detectable through direct cues

We categorized analyses based on scale of assessment and predictions concerning how coyotes respond to direct and indirect features associated with mountain lions and carrion at kills made by mountain lions. First, we implemented a spatially and temporally extensive analysis of habitat selection by coyotes that integrated both direct and indirect assessments of mountain lions and their kill sites (Figure 1). Direct and indirect responses to risk and reward are likely to occur at different spatiotemporal scales. For example, the range at which a source of risk or reward is directly perceivable should dictate the scale at which direct responses occur—for difficult to perceive sources of risk and reward, memory and learning may allow an animal to use indirect assessments to respond at greater scales than would be possible with direct assessments. Despite combining two potentially different scales of assessment (i.e., direct vs. indirect), our first analysis was consistent with contemporary methods of evaluating risk‐avoiding and reward‐seeking tactics (Davies et al., 2021). Importantly, we compared the results of this analysis with direct inferences drawn from analyses in the following two assessments. Analyses in the second and third assessments leveraged presumably stronger, more direct inferences (Prugh et al., 2019), to evaluate coyote behavior at discrete sources of risk and reward that vary in relative perceptibility. We evaluated movement and habitat selection by coyotes in response to direct encounters with mountain lions. Similarly, we used a direct encounter framework to evaluate the use of mountain lion kills by coyotes.

### 2.4 Direct versus indirect assessments of risk and reward

We used Random forest (RF) models (Breiman, 2001) to develop spatial predictions of probable mountain lion use (risk) and kill site occurrence (reward) across our study area (Huggler et al., in revision). We expanded on Huggler et al. (in revision)’s models by including kill sites containing elk and pronghorn to characterize the full suite of ungulate kill sites. Random forest is a machine learning approach that combines many classification trees, does not rely on normality, and can readily incorporate interactions and variables that are correlated (Breiman, 2001). We excluded investigated kill sites when prey were smaller than a juvenile mule deer (<1 year) to increase the likelihood that there were scavengeable resources available to coyotes. To increase our sample size of mountain lion kills with large prey present for our direct assessment, we used the methods described in Clapp et al. (in press) to generate predictions of kills made by mountain lions based on clusters of mountain lion locations. We used a receiver operating characteristic (ROC) curve and the subset of investigated kill sites containing large prey relative to investigated kill sites where we found small or no prey to develop a new cutoff for identifying kills of large prey that maximized sensitivity, while allowing for a 5% false positive rate. We compared the sensitivity metric across the four models implemented in Clapp et al. (in press) and found the Wilckens et al. (2016) model performed well for our application.

We used the RF predictions of mountain lion use and RF predictions of kill site occurrence, respectively, as indirect indices of risk and reward in our analysis. These layers represented the relative probability of occurrence for mountain lions and their kill sites based on the habitat features with which each are associated. We quantified direct metrics of risk and reward (for any given coyote location) by multiplying (1) the distance to each mountain lion location by the time since each mountain lion location, and (2) the distance to each mountain lion kill by the time since each mountain lion kill. We then selected the minimum value of each metric at each coyote location. We used the minimum value of distance multiplied by time to identify locations of risk and reward that are most spatiotemporally relevant.

We used integrated step‐selection functions (iSSFs; Avgar et al., 2016) and the *amt* package (Signer et al., 2019) within R (version 4.0.4; R Core Team, 2021) to evaluate whether the perceptibility of risk and reward influenced how coyotes navigate the landscape. Integrated step‐selection functions alleviate the potential bias of traditional SSFs by simultaneously allowing for the estimation of habitat selection and movement processes (Avgar et al., 2016; Forester et al., 2009). We used coyote locations during 15 May–30 September, when collars were programmed to acquire one location every hour (overall fix success = 89.8%). Consistent with other studies (Davies et al., 2021; Prokopenko et al., 2017), we paired each observed step with 10 available steps, which should adequately meet sampling requirements without causing computational limitations (Northrup et al., 2013; Thurfjell et al., 2014). We sampled available step lengths from a gamma distribution parameterized from the observed step lengths used by coyotes (shape = 0.38, scale = 1402.57), and available turning angles in radians from values between –*π* and *π* following a uniform distribution. We included fixed effects for step length and the natural logarithm of step length to reduce inferential bias in modeling habitat selection (Forester et al., 2009), and tested for multicollinearity with all predictor variables, removing the variable with the highest score until all were <3 VIF (Zuur et al., 2007). We developed a base model (eqn. 1) with landscape variables assessed at a 30‐m resolution expected to influence coyote habitat selection including: distance to roads, distance to aspen, distance to forest, elevation, Topographic Position Index (TPI; De Reu et al., 2013), Terrain Ruggedness Index (TRI; Riley et al., 1999), and fractional components of shrub height, bare ground, herbaceous, and sage height (Rigge et al., 2020). The model was clustered by coyote ID with the robust standard error estimator available within the *survival* R package (Therneau, 2020) and all predictor variables were centered and scaled by subtracting the mean and dividing by the standard deviation of each variable before modeling.

(1) $$\begin{array}{c} w\left(x\right)∼\text{StepLength}+\ln\left(\text{StepLength}\right)+\text{DistRoads}+\text{DistAspen}+\text{DistFor}\\ +\text{Elev}+\text{TPI}+\text{TRI}+\text{ShrubHeight}+\text{BareGround}+\text{Herb}+\text{SageHeight} \end{array}$$

We tested the base model against four additional models (Equations (2), (3), (4), (5)), with added combinations of direct and indirect metrics of risk and reward. We calculated the natural logarithm of direct metrics to account for a reduced effect at larger spatiotemporal scales. To test the prediction that direct assessments would be favored for carrion at kills made by mountain lions, whereas, indirect assessments would be favored for mountain lions, we ranked candidate models using the quasi‐likelihood under independence criterion (QIC), which considers independent clusters of observations and is appropriate for comparing models fit to autocorrelated data (Pan, 2001).

(2) $$w\left(x\right)∼\text{Eqn}1+\text{ln}\left(\text{DirectKill}\right)+\text{IndirectLion}$$

(3) $$w\left(x\right)∼\text{Eqn}1+\text{IndirectKill}+\text{IndirectLion}$$

(4) $$w\left(x\right)∼\text{Eqn}1+\ln\left(\text{DirectKill}\right)+\ln\left(\text{DirectLion}\right)$$

(5) $$w\left(x\right)∼\text{Eqn}1+\text{IndirectKill}+\ln\left(\text{DirectLion}\right)$$

### 2.5 Coyote–mountain lion encounters

We used locations from GPS collars to identify encounters between coyotes and mountain lions. We defined encounters as one location from a collared coyote and one from a collared mountain lion, occurring within 1 km and 2 h of one another while coyote collars were programmed to acquire hourly locations (15 May–30 September). We selected a 1‐km distance criteria to maintain sample size in analyses and because other studies have evaluated interactions involving apex predators at scales equal to, or coarser than 1 km (Broekhuis et al., 2013, 2019; Creel et al., 2008; Liley & Creel, 2008; Middleton et al., 2013). Although a 1‐km distance may be relatively coarse for identifying encounters with a stalk‐and‐ambush predator, we selected this distance in part not only due to sample size limitations but also so that we could test conventional methods of evaluating predator–prey encounters (e.g., Middleton et al., 2013). Encounters between two animals should be more likely to occur when both are active (Avgar et al., 2008; Scharf et al., 2006), and previous research in our study area indicated that activity curves for coyotes and mountain lions have a high degree of overlap (Huggler et al., in revision). Thus, while mountain lions use a more stationary hunting strategy than coyotes, they are unlikely to be entirely stationary in the time leading up to encounters. Mountain lion movement in combination with the relative openness of habitat in our study area (i.e., forested sections are small and rarely used by coyotes) should increase the distance at which coyotes are able to detect mountain lions, such that 1 km should not be an unreasonable detection distance. We used a 2‐h window to define encounters to reduce the number of missed encounters caused by differing fix rates between coyotes and mountain lions. Encounters often spanned several hours (i.e., after an encounter began, successive coyote and mountain lion locations remained within 1 km of each other). We classed encounters as ended when coyote and mountain lion locations were no longer within 1 km and 2 h of each other. We only evaluated coyote behavior in the time before an encounter began and after it ended because we were interested in pre‐ and post‐encounter behavior. We also excluded encounters that occurred within five hours of a previous encounter to ensure encounters were reasonably independent of one another.

We used a Generalized Additive Mixed Model (GAMM) within the *mgcv* R package (Wood, 2017) to test the effect of time to encounter in the five hours pre‐ and post‐encounter on hourly movement rate of coyotes (m/h). We modeled movement rate using a gamma error distribution, while accounting for time of day, and including nested random effects for coyote ID and encounter ID.

We evaluated shifts in habitat selection and turning angle by coyotes using an iSSF in the ten hours before and after encounters. Based on our movement rate analysis (aforementioned), ten hours encompassed the period in which pre‐ and post‐encounter behavior was likely to change. We paired each observed step with 10 available steps (Davies et al., 2021; Northrup et al., 2013; Prokopenko et al., 2017; Thurfjell et al., 2014) sampled from distributions of step lengths and turning angles, with step lengths generated separately for pre‐ and post‐encounter groups to preserve existing differences in movement behavior. We sampled turning angles in radians for available steps from a uniform distribution ranging from –*π* to *π*, and sampled step lengths from gamma distributions parameterized from the observed step lengths used by coyotes (pre‐encounter: shape = 0.38, scale = 1177.75; post‐encounter: shape = 0.41, scale = 1413.39).

We used conditional logistic regression clustered by coyote ID to estimate coyote step selection based on coyote turning angle, and on landscape variables expected to influence the risk of coyote predation. We assessed habitat selection for escape cover by dividing the landscape into three cover classes assessed at a 30‐m resolution: open (reference category), shrubs >0.5 m in height, and trees (LANDFIRE, 2016). We categorized shrubs that were ≤0.5 m as “open” because they were not tall enough to conceal an average‐sized coyote (Hinton & Chamberlain, 2014; Hinton et al., 2019). We calculated the proportion of each cover type occurring within a buffer at the end of coyote steps. We selected a buffer diameter of 256 m by dividing the average coyote step length by two. We included Terrain Ruggedness Index (TRI; Riley et al., 1999) as an additional cover covariate, and included distance to mountain lion, a metric of the distance in meters to the most recent mountain lion location at the end of each step. Similar to analyses mentioned previously, we included fixed effects for step length and the natural logarithm of step length and reduced multicollinearity, when present, by removing the variable with the highest score until all were <3 VIF (Zuur et al., 2007). Finally, we added interactions of each fixed effect excluding step length and the natural logarithm of step length with time to encounter in the 10 h pre‐ and post‐encounter, and centered and scaled predictor variables by subtracting the mean and dividing by the standard deviation of each variable before modeling.

### 2.6 Kill site use by coyotes

**FIGURE 2:**
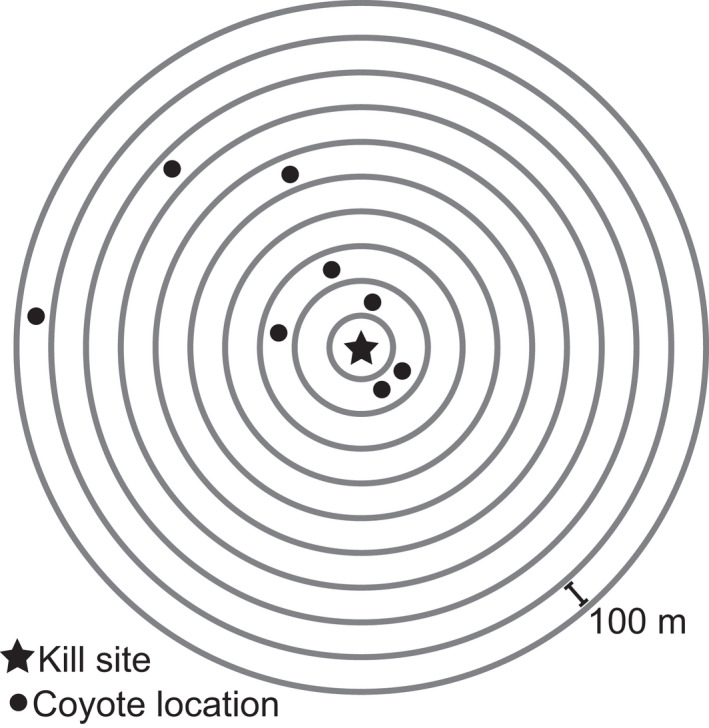
Conceptual schematic showing concentric rings surrounding kills made by mountain lions (*Puma concolor*) with radii ranging from 100 to 1000 m. We assessed the probability of coyote (*Canis latrans*) use at predicted kills made by mountain lions by classifying rings as used in the two weeks before and after the initial mountain lion location at a kill site

We used GAMMs to model the probability of use by coyotes at kills made by mountain lions as a function of days, and distance from the kill, using the combined investigated and predicted kills of large prey (outlined previously; “*Direct versus Indirect Assessments of Risk and Reward”*). We modeled the probability of coyote use separately during the two weeks before and after the first mountain lion location at each kill site. We selected two weeks post‐kill as our observation window based on the rate of carrion removal we observed during investigations of kills made by mountain lions (>95% carcass removal within two weeks). To ensure each kill site was reasonably accessible to coyotes, we excluded kill sites that did not have coyote locations within the 1000‐m radius surrounding the kill within two weeks of the first mountain lion location. We determined use based on the presence of a coyote location in the space between 10 concentric circles with radii ranging from 100 m to 1000 m on each of the 14 days pre‐ and post‐kill (Figure 2). We considered a kill site inactive based on the last mountain lion location occurring at the kill site, and included a categorical predictor describing whether the mountain lion was still using the kill in the post‐kill model to examine if mountain lions deterred use by coyotes. We also incorporated a count of coyote locations in the surrounding 2500 m radius (adjusted for collar fix rate) as a fixed effect in both models to account for underlying variation in probability of use based on sampling. Finally, we added offsets for the inverse of collar fix rate and the area of the concentric ring and included random effects for kill site ID. We tested for multicollinearity (<3 VIF; Zuur et al., 2007) and performed backwards stepwise selection to eliminate variables using Akaike's Information Criterion (AIC) (Akaike, 1974).

## 3 RESULTS

### 3.1 Direct versus indirect assessments of risk and reward

**FIGURE 3:**
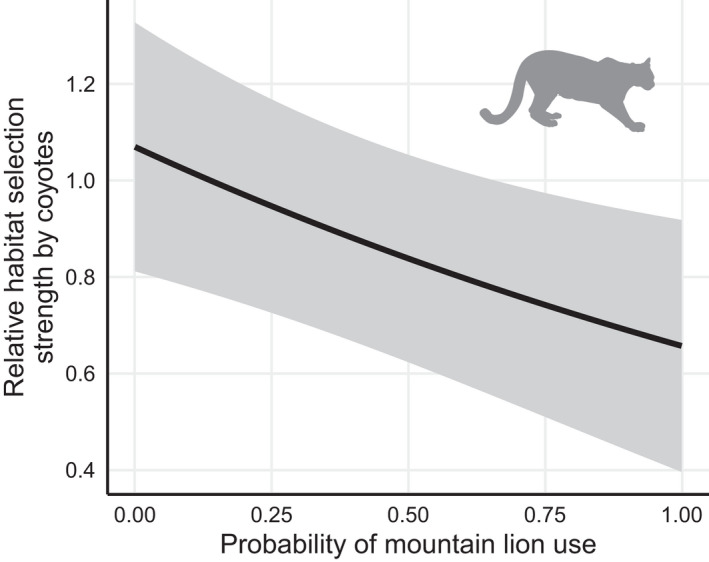
Relative habitat selection strength (exponentiated linear predictor) for coyotes (*Canis latrans*), relative to the probability of use for mountain lions (*Puma concolor*) with 95% CI. Probability of use for mountain lions represents an indirect assessment of mountain lion presence based on the habitat features used by mountain lions. Predicted from an integrated step‐selection function modeling coyote movement based on indirect assessments of risk and reward using coyote locations occurring during May 2017–September 2020 in southwestern Wyoming, USA

We found support for two models relative to the base habitat model when assessing whether coyotes exhibit selection or avoidance for the presence of mountain lions and mountain lion kills (*n* = 398) based on direct or indirect assessments of their presence on the landscape. Only the hypothesized model, which integrated direct kill and indirect mountain lion assessments (ln(DirectKill) + IndirectLion; Equation 2), and the indirect‐only model (IndirectKill + IndirectLion; Equation 3) improved QIC scores relative to the base model, with the indirect‐only model ranked highest (Table 1). The mountain lion and kill site locations selected to calculate direct metrics of risk and reward were on average temporally close (mountain lions: mean = 0.14 days, range = 0.02–1205 days; kill sites: mean = 4.79 days, range = 0.02–1258 days), and spatially distant (mountain lions: mean = 17.65 km, range = <0.001–77.67 km; kill sites: mean = 20.87 km, range = 0.005–77.80 km) from observed coyote locations. Indirect assessments of mountain lion use drove model performance beyond the base model for the model including direct‐kill and indirect‐mountain lion metrics, as well as the indirect‐only model (Figure 3), and neither kill site metrics (direct or indirect) were significant predictors of coyote step selection (Table 2).

**TABLE 1:** Model performance statistics for iSSFs of coyote (*Canis latrans*) habitat selection under varying assessments of mountain lions (*Puma concolor*; risk) and kills made by mountain lions (reward) relative to a base habitat model

Model	Quasi‐LL	*K*	ΔQIC
Base + IndirectKill + IndirectLion	−394335.1	14	0.0
Base + ln(DirectKill) + IndirectLion^a^	−394346.0	14	6.7
Base	−394401.1	12	65.7
Base + ln(DirectKill) + ln(DirectLion)	−394400.0	14	79.3
Base + IndirectKill + ln(DirectLion)	−394400.2	14	110.3

Note

The following are provided for each model: quasi‐likelihood (Quasi‐LL), number of predictors (*K*), difference in QIC between model and best performing model (ΔQIC). Direct metrics were quantified based on the distance‐to and time‐since locations of risk and reward, whereas indirect metrics were quantified based on predictions of the probability of occurrence for risk and reward based on habitat associations. Coyote, mountain lion, and kill site data collected during May 2017–September 2020 in southwestern Wyoming, USA.

^a^ Hypothesized highest ranked model based on the low perceptibility of mountain lions and high perceptibility of kills made by mountain lions.

**TABLE 2:** Model summary for the highest ranked model (IndirectKill + IndirectLion; eqn. 3) from the iSSFs of coyote (*Canis latrans*) habitat selection and movement under varying assessments of mountain lions (*Puma concolor*; risk) and kills made by mountain lions (reward)

Predictor	*β*	*SE*	*z*	*p*
StepLength	−0.001	0.018	−0.051	.959
**ln(StepLength)**	**0.047**	**0.015**	**3.145**	.**002**
IndirectKill	0.019	0.014	1.303	.193
**IndirectLion**	**−0.056**	**0.020**	**−2.861**	.**004**
**BareGround**	**−0.175**	**0.043**	**−4.087**	**<.001**
**Herb**	**0.117**	**0.024**	**4.963**	**<.001**
SageHeight	0.004	0.014	0.282	.778
**ShrubHeight**	**0.077**	**0.017**	**4.521**	**<.001**
DistRoads	0.004	0.021	0.170	.865
DistAspen	−0.091	0.112	−0.813	.417
DistFor	0.015	0.065	0.230	.818
Elev	−0.184	0.107	−1.723	.085
**TPI**	**−0.039**	**0.011**	**−3.509**	**<.001**
**TRI**	**0.051**	**0.025**	**2.049**	.**040**

Note

Covariates include habitat variables, movement parameters, and indirect assessments of mountain lions and their kills. Indirect metrics were quantified based on predictions of the probability of occurrence for risk and reward based on habitat associations. The following are provided for each predictor: beta coefficient (*β*), robust standard error estimate (*SE*), *z*‐score (*z*), p‐value (*p*). Coyote, mountain lion, and kill site data collected during May 2017–September 2020 in southwestern Wyoming, USA. Boldface indicates variables where *p* < .05.

### 3.2 Coyote–mountain lion encounters

**FIGURE 4:**
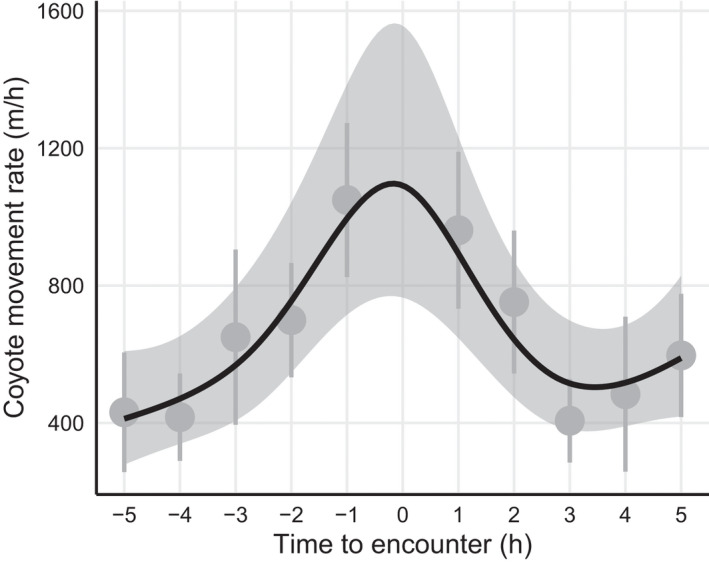
Predicted coyote (*Canis latrans*) movement rate (meters/hour) and 95% CI bounds (light gray) in the five hours pre‐ and post‐encounter with mountain lions (*Puma concolor*). Predicted movement rate is overlaid on average hourly coyote movement rate (dark gray with 95% CIs). Peaks in the movement rate of prey (coyotes) may coincide with, and ultimately drive encounters with a less mobile stalk‐and‐ambush predator. We modeled movement rate based on *n* = 54 encounters with mountain lions involving *n* = 15 coyotes occurring during May 2017–September 2020 in southwestern Wyoming, USA, after accounting for variation in movement rate caused by time of day

We identified 54 independent encounters occurring between 15 coyotes and 4 mountain lions. Of these, the majority of encounters (57.6%) spanned multiple hours, rather than occurring at a single point in time. Movement rate of coyotes varied as a function of time to encounter with a mountain lion (E.D.F. = 3.671, *p* < .001) and time of day (E.D.F. = 5.456, *p* < .001). Despite time to encounter being a predictor of movement rate, movement rate did not increase post‐encounter in a manner consistent with coyotes fleeing from mountain lions. Movement rate increased as encounters neared, peaked immediately before the encounter, and decreased after the encounter (Figure 4). The fitted curve for time to encounter matched closely with the average hourly movement rates of coyotes (Figure 4). In the hour before and after encounters, movement rate was 1049 m/h (95% CI = 825–1273) and 961 m/h (95% CI = 733–1190), respectively, compared with 417 m/h (95% CI = 290–544) and 484 m/h (95% CI = 258–710) four hours pre‐ and post‐encounter. Similar to movement rate, we discovered no pattern consistent with coyotes altering their selection of habitat after an encounter with mountain lions. Selection of habitat remained similar pre‐ and post‐encounter (Table 3).

**TABLE 3:** Model summary from the iSSF model of coyote (*Canis latrans*) habitat selection and movement behavior across time to encounter from direct encounters with mountain lions (*Puma concolor*)

Predictor	*β*	*SE*	*z*	*p*
StepLength	0.030	0.062	0.490	.624
ln(StepLength)	−0.010	0.035	−0.295	.768
cos(TurnAngle)	−0.012	0.032	−0.368	.713
**Shrub**	**0.203**	**0.091**	**2.217**	.**027**
Tree	0.024	0.090	0.267	.790
**DistLion**	**−0.608**	**0.088**	**−6.935**	**<.001**
TRI	−0.054	0.061	−0.892	.372
cos(TurnAngle):Time2Enc	0.024	0.034	0.686	.493
Shrub:Time2Enc	−0.022	0.037	−0.578	.563
Tree:Time2Enc	0.005	0.056	0.094	.925
**DistLion:Time2Enc**	**0.467**	**0.117**	**3.976**	**<.001**
TRI:Time2Enc	−0.029	0.022	−1.319	.187

Note

Covariates include cover attributes, movement parameters, and distance to the nearest mountain lion. The following are provided for each predictor: beta coefficient (*β*), robust standard error estimate (*SE*), z‐score (*z*), *p*‐value (*p*). Coyote and mountain lion location data collected during May 2017–September 2020 in southwestern Wyoming, USA. Boldface indicates variables where *p *< .05.

**FIGURE 5:**
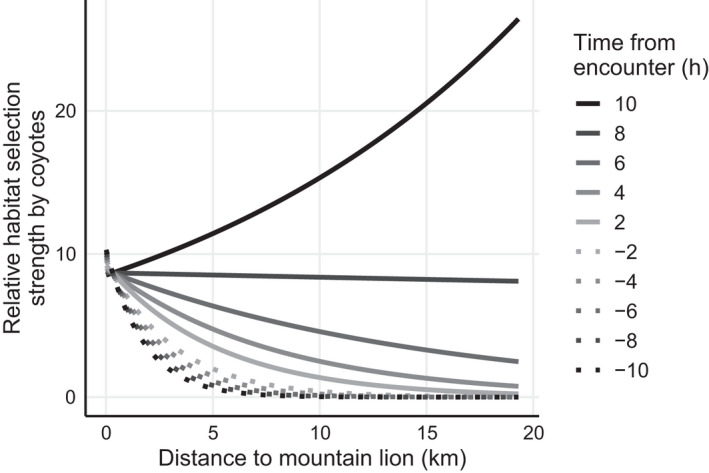
Relative habitat selection strength (exponentiated linear predictor) for coyotes (*Canis latrans*), predicted from an integrated step‐selection function modeling coyote movement characteristics and habitat selection around *n* = 54 encounters with mountain lions (*Puma concolor*) involving *n* = 15 coyotes occurring during May 2017–September 2020 in southwestern Wyoming, USA. We calculated distance to the most recent mountain lion location at each step and interacted that with hourly time to encounter in the 10 h pre‐ and post‐encounter

Consistent with nonfleeing behavior, coyotes did not show selection for turning angle as a fixed effect or when interacted with time to encounter (Table 3). Coyotes altered their selection for distance to mountain lions across time to encounter (Table 3), but not in the manner associated with fleeing behavior. Because a coyote and mountain lion must move closer together to initiate an encounter, and move further apart to terminate an encounter, we expected that coyotes would show selection for reduced distance to mountain lions as an encounter approaches and farther from mountain lions immediately after the encounter. Instead, coyotes only began to select for increased distance from mountain lions after 9–10 h had passed after the encounter (Figure 5).

### 3.3 Kill site use by coyotes

**FIGURE 6:**
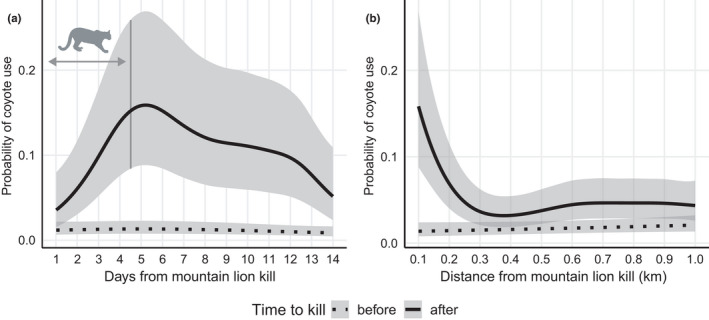
Probability of coyote (*Canis latrans*) use at predicted kills made by mountain lions (*Puma concolor*) and shaded 95% CIs for two weeks before (dotted line) and after (solid line) the kill. (a) The probability of use across the 14 days on either side of the kill, where the *x*‐axis extends across 14 days pre‐kill for the “before” line and 14 days post‐kill for the “after” line. The vertical dark gray line represents the average time spent at kill sites by mountain lions in the time after the kill was made (mean = 4.50 days, 95% CI = 4.35–4.66, IQR = 2.50–5.47). (b) The probability of use assessed within concentric rings surrounding kills with radii ranging from 100–1000 m. Coyote and mountain lion kill site data collected during May 2017–September 2020 in southwestern Wyoming, USA

Collared coyotes were present in the 1000‐m area surrounding 33 of the 398 mountain lion kills in the two weeks before the kill, and were present at 30 kill locations in the two weeks afterwards. Mountain lion feeding status was removed from the post‐kill model based on backwards elimination (ΔAIC = 183.2 relative to the reduced model), suggesting lion presence was not a primary determinant of coyote scavenging. In the pre‐kill model, neither distance from the kill (E.D.F. = 1.000, *p* = .139), nor days from the kill (E.D.F. = 1.864, *p* = .185) were statistical predictors of the probability of coyote use, and probability of use was minimal (Figure 6a). Probability of coyote use at kills made by mountain lions varied as a function of distance from the kill (E.D.F. = 4.528, *p* < .001) and days from the kill (E.D.F. = 4.559, *p* < .001) in the post‐kill model. The probability of coyote use increased initially with days from the mountain lion kill, peaking just after the average time mountain lions were active at kill sites (mean = 4.50 days, 95% CI = 4.35–4.66, IQR = 2.50–5.47), and decreasing thereafter (Figure 6a). Coyote use was greatest within the 100‐m radius surrounding kill sites in the post‐kill model and decreased dramatically with increased distance from the kill between the 100‐ and 300‐m radii (Figure 6b).

## 4 DISCUSSION

Our work extended conventional questions within predator–prey systems to a predator–predator context to assess how a mesopredator navigates the risk–reward landscape created by an apex predator. We found partial support for our first hypothesis in that coyotes demonstrated avoidance toward the habitat used by mountain lions (i.e., indirect assessment), but did not exhibit selection or avoidance toward kill sites. Coyotes did not exhibit fleeing behavior, nor did they seek cover, following spatiotemporal encounters with mountain lions. These results are consistent with our hypothesis that coyotes may be limited in their ability to detect an ambush predator like a mountain lion, which is a pattern that likely will emerge in other predator–prey systems that include an ambush predator. Despite the risk imposed by mountain lions (i.e., coyotes composed 8.4% of mountain lion diets in our study area), coyotes successfully (i.e., no GPS‐collared coyotes were killed during the time spent actively scavenging at kills made by collared mountain lions) exploited mountain lion kills shortly after the kill was established. The initial increase in the pattern of use of mountain lion kills by coyotes coincided with a discovery phase—an outcome in support of our final hypothesis. Coyotes navigated and made use of mountain lion kills directly, which is consistent with apex predators provisioning food resources for subordinate carnivores (Elbroch & Wittmer, 2012; Prugh & Sivy, 2020; Ruprecht et al., 2021; Sivy et al., 2017, 2018). Our work supported both direct and indirect assessments when characterizing the navigation of risk and reward by mesopredators, but support for each assessment was largely dependent on perception, scale, and sampling limitations.

### 4.1 Methodological insights

In our comprehensive iSSF analysis comparing direct and indirect assessments, coyote step selection was best explained when the presence of mountain lions and kill sites were assessed indirectly. In support of our prediction, coyotes avoided mountain lions through indirect assessment of their presence, but in contrast to our expectations, coyotes failed to assess kill sites directly. Notably, neither kill site metrics (direct or indirect) were statistically significant predictors of habitat selection by coyotes in our iSSF analysis, despite clear patterns of use at kills made by mountain lions in our latter analyses. Although our findings contradict recent guidance toward using direct assessments within analytical frameworks (Prugh et al., 2019), we believe there are a number of factors that led to our inability to detect the use of mountain lion kills by coyotes under a step‐selection approach. Rather than coyote indifference toward the reward of carrion left at mountain lion kill sites, it is more likely that the temporal scale of our data limited our ability to identify direct navigation within our iSSF analysis. For any source of risk or reward, there should be a spatiotemporal threshold where direct perception becomes possible. Based on the greater perceptibility of carrion relative to mountain lions, we expected that direct navigation toward kill sites would occur at a greater scale, and accordingly, we expected direct navigation toward those sources would be easier to detect within our iSSF than that away from the risk of mountain lions. Despite likely occurring at a greater scale, we were still not able to capture the direct perception of coyotes toward kills made by mountain lions. In a recent study where direct and indirect responses were framed as “reactive” and “proactive,” respectively, African wild dogs (*Lycaon pictus*) avoided the risk of predation from African lions (*Panthera leo*) using indirect rather than direct responses to risk when both were compared within iSSF analyses (Davies et al., 2021). As is indicated by the framing of direct responses as “reactive”, direct responses are perhaps more likely to occur at finer spatiotemporal scales than indirect responses. The absence of detecting a direct response in this context should not necessarily be considered as evidence for the lack of a meaningful direct response; instead, the absence of a direct response may be indicative of mismatched and inappropriately addressed differences in scale. Habitat selection analyses that fail to address scale limitations will likewise fail to accurately assess the responses occurring at finer scales, and researchers must carefully consider scale when comparing the movement of one animal with respect to another.

In movement and habitat selection analyses, indirect assessments of risk and reward may be favored when data characterizing the true arrangement of those sources on the landscape is limited. Indirect assessments fill spatial gaps in data caused by sampling error through habitat associations that can be extended across the landscape (Mumma, Gillingham, et al., 2017). We suspect the presence of unsampled kill sites limited our ability to test for direct selection. Moreover, if unsampled kill sites were spatially predictable (i.e., occurred within habitats associated with mountain lion kills), we would have interpreted selection for those areas as navigation based on indirect assessment, even if coyotes actually identified those kills based on direct cues. The presence of unsampled kill sites combined with relatively coarse GPS fix rates (hourly) likely attenuated responses, ultimately leading to weak evidence of direct selection by coyotes for kill sites. Limitations in perception, scale of assessment, and sampling error may reduce the effectiveness of direct assessments in broad‐scale movement and habitat selection analyses, however, when those issues are addressed, direct assessments may greatly improve ecological inference.

Confounding effects can make it difficult to determine if selection for a habitat feature is a true response to its presence on the landscape (Northrup et al., 2012; Roever et al., 2010). Similarly, avoidance of the habitat selected by another species is not necessarily avoidance of that species; instead, it may simply be a reflection of differing habitat niches (Mumma, Holbrook, et al., 2017). Habitat selection does not directly communicate the particular cues an animal uses to navigate its environment and does not necessarily indicate that the pattern in habitat selection is a direct result of the habitat feature tested. As such, it is difficult to conclude that indirect avoidance of the habitat used by mountain lions is a conscious avoidance of mountain lions by coyotes. Instead, coyotes may simply be selecting for habitat that differs from mountain lions because they are different predators with different life histories and hunting tactics. While it is likely that coyotes avoid mountain lions since mountain lions represent a mortality risk, determining this with certainty is difficult. By recognizing these limitations and nuances, researchers are likely better prepared to implement analyses that appropriately leverage data to look at direct interactions occurring at finite points in space and time.

Had we only performed our integrated step‐selection analysis to evaluate direct and indirect assessments of risk and reward by coyotes, we would have incorrectly concluded that coyotes do not use carrion left at kills made by mountain lions. The striking contrast in conclusions drawn from each of our analyses highlights the importance of evaluating species interactions from multiple angles. Although implementing broad‐scale habitat selection analyses is appealing because they allow researchers to answer questions regarding selection and movement processes around a variety of habitats and species simultaneously, they may cloud much of the nuance we are intending to understand. In our study, there was more nuance in the navigation of risk and reward than could be detected through a comprehensive step‐selection analysis. By using a direct assessment with discrete locations to test the use of mountain lion kills by coyotes, we were able to more effectively use our data to assess the responses to immediate risk and reward.

### 4.2 Mesopredator response to apex predator

Coyote movement behavior and habitat selection following encounters with mountain lions suggested that coyotes are likely limited in detecting a nearby stalk‐and‐ambush predator. In typical predator–prey assessments, movement rate of the prey reaches a maximum post‐encounter; however, in many of these interactions, the predator is cursorial and causes encounters through their pursuit for prey (Middleton et al., 2013). In encounters with stalk‐and‐ambush predators, the predator may be less likely to create encounters through its own movement. Instead, it appears coyotes may facilitate encounters with mountain lions by increasing their movement rate and unknowingly moving into risky spaces. Despite mountain lions sometimes exhibiting individual specialization in their pursuit for particular prey (Lowrey et al., 2016), the movement of animals they hunt also must play a part in facilitating encounters. Indeed, the underlying probability of a spatial encounter between a stationary object and a moving object will increase with the speed of the moving object, and the same pattern will follow in encounter frequencies between mobile prey and an ambush predator (Avgar et al., 2008; Scharf et al., 2006). The degree to which prey facilitate encounters should be affected by the degree of locomotion employed by the predator—at the extreme, if an apex predator remains motionless for days waiting for prey to move within striking distance, encounters may be almost entirely dependent on prey movement. In predator–prey or apex predator–mesopredator systems involving an ambush apex predator, these ideas should be fundamental to informing expectations about spatiotemporal responses. Future work should evaluate the degree to which prey movement rate dictates the rate of encounters with stalk‐and‐ambush predators. In our study, mountain lions were not likely stationary through the entirety of the 5 h we investigated prior to encounters; however, they likely were using a more stationary movement strategy than coyotes. Consequently, encounters between coyotes and mountain lions likely occurred in part because of increased movement and activity by coyotes.

The absence of a response by coyotes to encounters with mountain lions does not mean that coyotes are incapable of detecting a nearby mountain lion—there are limitations in our ability to detect responses to encounters with mountain lions due to the scale of our assessment. For instance, at the scale of meters and seconds, coyotes almost certainly respond to the presence of mountain lions, or perhaps would be killed. Our work indicates that at the scale of our sampling, coyotes are likely limited in their ability to detect mountain lions directly relative to that seen in other predator–prey studies, and similarly, coyotes may not directly perceive mountain lions during typical encounters. We assessed encounters within a 2‐hour temporal window to handle sample size limitations exacerbated by differing fix rates between coyotes and mountain lions. Despite assessing encounters at a coarser temporal scale than has been implemented in similar studies (Middleton et al., 2013; Oates et al., 2019), we believe this had minimal effect on our ability to detect behavioral responses of coyotes to encounters with mountain lions. Encounters between coyotes and mountain lions often occurred across a sequence of consecutive locations, meaning both species remained within a 1‐km distance for several hours. These instances, coupled with the lack of coyote response, provided further evidence that coyotes may be unaware of nearby mountain lions.

We defined our encounter criteria at a spatial scale consistent with that shown to elicit behavioral responses in other studies involving large mammalian predators and prey (Broekhuis et al., 2013; Creel et al., 2008; Liley & Creel, 2008; Middleton et al., 2013; Oates et al., 2019). Even when responses have only occurred at finer spatial scales, low perceived risk has been implicated as the causal mechanism, rather than a failure to perceive the predator itself (Broekhuis et al., 2019; Oates et al., 2019). In our study, coyotes did not respond to the presence of mountain lions, despite being vulnerable to predation. Because the perception of risk does not necessarily lead to a behavioral response (Gaynor et al., 2019), it is possible that coyotes perceived nearby mountain lions, but either did not consider them to be a source of risk, or did not perceive the immediate risk they imposed to be sufficient enough to justify a response. In the absence of cause‐specific mortality data for this study, an explanation for the nonresponse of coyotes to mountain lions is that we have overestimated the degree of risk, and that coyotes may not actually consider mountain lions risky. While the composition of the mountain lion diet suggests that the risk to individual coyotes can be high, it is difficult to conclude with certainty that the same level of risk applies to the entire population. Similarly, scavenging by coyotes at kills made by mountain lions suggests that coyotes may not perceive mountain lions as a substantial source of risk; although, in this context there is a significant reward (i.e., carrion) present, likely influencing the risk–reward assessment by coyotes. A hypothesis of fatal attraction has been proposed to explain opposing positive local effects and negative landscape‐scale effects of wolves on mesopredators (Sivy et al., 2017). The hypothesis implies that mesopredators may be somewhat unaware of the extent of predation risk they face by scavenging carrion left by apex predators (i.e., an ecological trap) (Prugh & Sivy, 2020). Our findings indicate that the risk associated with the act of scavenging itself may be relatively low. At investigated kill sites, coyotes were accompanied by other prey only 13.6% of the time and, therefore, in the majority of instances, coyotes were not scavenging when they were killed by a mountain lion. Additionally, although mountain lions in our study likely killed GPS‐collared coyotes, collared coyotes were never killed while actively scavenging at kills made by collared mountain lions. Coyotes may ameliorate predation risk at kills made by mountain lions by exhibiting increased vigilance (Young & Mahoney, 2017), by exploiting different behavioral states and windows in the time spent at kills by mountain lions, or by arriving after a mountain lion has stopped feeding at its kill. Alternatively, mountain lions may be less likely to attack coyotes when they have recently killed and consumed other prey (Ruprecht et al., 2021).

Recent reviews have highlighted the importance of scavenging in structuring communities, and the knowledge gaps regarding the interactions among predators driven by carrion provisioning (Moleón et al., 2014; Prugh & Sivy, 2020). If risk at kill sites of apex predators is low, scavenging is appealing because it allows mesopredators to access resources without the need to catch their own prey (Wilson & Wolkovich, 2011). In our study, coyotes perceived and scavenged carrion left at kills made by mountain lions, and appear to have done so without increasing their risk of predation. The extent of scavenging employed by a predator should vary based on the resources available, and the degree to which scavenging is facultative versus obligate (Pereira et al., 2014). When scavenging becomes increasingly important (i.e., limited prey, obligate scavenger), and when the presence of carrion is increasingly predictable, a scavenger may be more likely to use indirect assessments to locate resources. For instance, brown hyenas (*Hyaena brunnea*) will travel great distances in search of carrion, and navigate along shorelines where they are more likely to encounter washed up carcasses (Mills, 1990). In contrast, when scavenging is less important, and when carrion is more dispersed, a mesopredator may be more likely to focus on finding its own prey but also exploit direct cues to locate carrion when available. We believe the increasing perceptibility of kill sites best explains coyote use because olfactory cues and other direct cues, such as visual detection of avian scavengers, should increase shortly after the kill, which corresponds precisely with the pattern of increased use we observed with coyotes. Other work has indicated that even for a highly concentrated and predictable resource, the absence of direct cues can dramatically decrease the use of an area by scavengers (Natusch et al., 2017). Thus, it is possible that in many systems, neither memory of the particular areas where resources were located nor associating resources with their related habitats are used alone to locate resources.

### 4.3 Conclusions

Our work demonstrates the nuance of interactions between apex predators and mesopredators, and begins to uncover the way in which mesopredators navigate benefits and costs associated with those interactions. We encourage holistic study designs and integration of analyses that directly test questions regarding selection for features of risk and reward across landscapes. Our work adds to the growing literature suggesting apex predators aid in food provisioning for subordinate carnivores (Elbroch & Wittmer, 2012; Prugh & Sivy, 2020; Ruprecht et al., 2021; Sivy et al., 2017, 2018), and provides new insights on expectations regarding prey movement rate and the corresponding rate of encounters with stalk‐and‐ambush predators. Broadly, our insights suggest that mesopredators may be capable of minimizing risky encounters with apex predators while also taking advantage of carrion resources available at kill sites by keying into spatial variation in habitat, and leveraging strong senses (e.g., olfactory, visual) targeted toward direct cues. Different species of mesopredators likely use differing perception tactics to identify sources of risk and reward, exploiting their unique strengths. Future work incorporating multiple mesopredators with presumably varying behavioral states, and perception strengths would advance our understanding of how mesopredators navigate the risk–reward landscape created by apex predators.

## CONFLICT OF INTEREST

The authors have no competing interests.

## AUTHOR CONTRIBUTIONS

**Mitchell J. Brunet:** Conceptualization (equal); Data curation (equal); Formal analysis (lead); Writing – original draft (lead); Writing – review & editing (lead). **Kevin L. Monteith:** Conceptualization (equal); Data curation (lead); Funding acquisition (lead); Writing – review & editing (equal). **Katey S. Huggler:** Data curation (lead); Writing – review & editing (supporting). **Justin G. Clapp:** Data curation (lead); Writing – review & editing (supporting). **Daniel J. Thompson:** Data curation (lead); Writing – review & editing (supporting). **Patrick W. Burke:** Data curation (equal); Writing – review & editing (supporting). **Mark Zornes:** Data curation (equal); Writing – review & editing (supporting). **Patrick Lionberger:** Data curation (equal); Writing – review & editing (supporting). **Miguel Valdez:** Data curation (equal); Writing – review & editing (supporting). **Joseph D. Holbrook:** Conceptualization (equal); Data curation (equal); Formal analysis (equal); Funding acquisition (equal); Writing – review & editing (equal).

## Data Availability

Data used for the analyses in this manuscript are accessible on Dryad (DOI https://doi.org/10.5061/dryad.v15dv41xh).
